# Electrospun Phage-Loaded Bilayer Nanofibrous Scaffolds for Wound Dressing Applications: A Comparative Study of Different Bacteriophages

**DOI:** 10.3390/jfb17020060

**Published:** 2026-01-26

**Authors:** Siavash Aghili, Muhammed Awad, Md Hasib Adnan, George Bouras, Tran Thanh Tung, Sarah Vreugde, Dusan Losic

**Affiliations:** 1 School of Chemical Engineering, Adelaide University, Adelaide, SA 5005, Australia; siavash.aghili@adelaide.edu.au (S.A.); tran.tung@adelaide.edu.au (T.T.T.); 2 Department of Surgery-Otolaryngology Head and Neck Surgery, Basil Hetzel Institute for Translational Health Research Woodville South, Adelaide University, Adelaide, SA 5011, Australiamdhasib.adnan@adelaide.edu.au (M.H.A.); sarah.vreugde@adelaide.edu.au (S.V.)

**Keywords:** electrospinning, phages, phage therapy, wound dressing, wound healing, antibacterial efficacy

## Abstract

Antimicrobial resistance is a growing global health threat, necessitating alternatives to conventional antibiotics. Bacteriophages, viruses that specifically target bacteria, represent a promising option, and phage-loaded electrospun fibers have recently gained attention as wound dressings for localized phage therapy. However, the influence of phage morphology and scaffold design has been largely overlooked. This study investigates how phage morphology and structure, in conjunction with scaffold design and processing conditions, may influence the biological performance of electrospun scaffolds. A bilayer scaffold was developed comprising a supportive polycaprolactone (PCL)/gelatin (70:30) layer and a polyvinyl alcohol (PVA) top layer loaded with bacteriophages. Two phage types, short-tailed podovirus APTC-SL.1 and long-tailed myovirus APTC-Efa.20, were incorporated into PVA fibers to evaluate their antibacterial activity against *Staphylococcus lugdunensis* and *Enterococcus faecalis*, respectively. The fibers were characterized using XRD, FTIR, TGA, optical microscopy, SEM, TEM, wettability analysis, and in vitro degradation tests. Biological assessments included antimicrobial testing, phage viability, and phage release. The bilayer scaffold containing short-tailed phages preserved phage viability and produced clear zones of lysis against *S. lugdunensis*, with ≈8.15% viability retained after electrospinning and relatively controlled release, whereas long-tailed phages showed no antibacterial activity. These results suggest that phage structure and morphology, together with electrospinning conditions and scaffold architecture, may play an important role in maintaining phage functionality in wound dressing applications, while acknowledging that host–phage interactions may also contribute to the observed differences.

## 1. Introduction

Antimicrobial resistance represents one of the most pressing global health threats, contributing to an estimated 4.95 million deaths worldwide in 2019 [1]. Among the clinically significant bacterial pathogens, *Staphylococcus aureus*, *Staphylococcus lugdunensis*, *Pseudomonas aeruginosa*, *Acinetobacter baumannii*, *Enterococcus faecalis* and *Klebsiella pneumoniae* are particularly concerning because of their increasing resistance to multiple antibiotics and their association with severe hospital- and community-acquired infections [2,3]. *S. lugdunensis* has emerged as an important opportunistic pathogen capable of causing a broad range of infections, including skin and soft tissue infections, wound infections, endocarditis, and osteomyelitis. Although traditionally regarded as a coagulase-negative *staphylococcus* with low virulence, accumulating evidence shows that *S. lugdunensis* exhibits pathogenic behavior comparable to *S. aureus,* including biofilm formation, production of cytolytic toxins, and adhesion to host tissues. The increasing incidence of antibiotic-resistant *S. lugdunensis* strains underscores the urgent need for new therapeutic strategies [4,5,6,7]. Bacteria acquire and disseminate resistance through several well-known mechanisms, such as enzymatic inactivation of drugs, active efflux of antimicrobial compounds, and modification of antibiotic targets via genetic mutation or horizontal gene transfer. The rapid evolution of these defence mechanisms limits the efficacy of conventional antibiotics and highlights the importance of exploring alternative antibacterial modalities [5,8,9].

One promising approach involves the use of bacteriophages (phages), viruses that specifically infect and lyse bacterial cells. During infection, lytic phages recognize receptors on the bacterial surface, inject their nucleic acid, and replicate intracellularly. Accumulation of phage progeny ultimately leads to bacterial cell lysis, releasing new viral particles to continue the infection cycle. Unlike broad-spectrum antibiotics, bacteriophages are highly specific to their target bacteria, leaving beneficial microbes unharmed and reducing the likelihood that resistance will develop in the microbial community [8,10,11,12]. In recent years, phage therapy has gained renewed attention as a viable alternative or adjunct to chemical antibiotics. Because phages are self-replicating and co-evolving entities, they maintain therapeutic effectiveness even as bacterial populations undergo genetic changes. Moreover, once the target bacteria are eradicated, residual phages degrade, minimizing adverse effects on host tissues [5,13,14,15].

Ensuring the viability and controlled release of bacteriophages at the infection site is essential for effective therapeutic application. For wound treatment, local delivery systems must conform to the wound surface, maintain biocompatibility, and preserve phage functionality [16]. Electrospun polymeric fibers offer an advantageous alternative to liquid or gel formulations, providing flexibility, high porosity, and controlled release of bioactive agents while minimizing tissue stress. The electrospinning process, which employs an electric field to produce ultrafine continuous fibers, is a simple and cost-effective method applicable to various natural and synthetic polymers. These nanofibrous materials have been explored for drug and antimicrobial delivery, wound dressings, and tissue engineering [17,18,19]. Notably, phages can be incorporated or bound to electrospun fibers, and electrostatic interactions have been shown to enable uniform distribution and gradual release from the matrix [20]. Thus, combining phage therapy with electrospun fiber technology represents a promising strategy for developing localized antibacterial delivery systems which is explored in several studies.

In one study, T4 bacteriophages were incorporated into electrospun polycaprolactone/collagen fibers to provide both antibacterial and hemostatic effects, yielding a multifunctional wound dressing with a potential for infection control and healing [21]. Sarhan and Azzazy [22] developed green electrospun honey, PVA, and chitosan nanofibers loaded with bee venom and bacteriophages to eliminate multidrug-resistant *P. aeruginosa* while promoting wound healing. Moreover, electrospun gelatin/silk fibroin nanofibers encapsulating phages were employed to achieve effective antibacterial activity against multidrug-resistant *P. aeruginosa* while maintaining phage viability and biocompatibility [23]. Vonasek et al. [20] and Nogueira et al. [24] investigated the immobilization of bacteriophages onto fibrous materials to develop effective antibacterial surfaces. Vonasek et al. [20] examined electrostatic interactions for immobilizing phages on cellulose microfibers, achieving uniform distribution, whereas Nogueira et al. [24] investigated covalent immobilization of the vB_Pae_Kakheti25 phage on polycaprolactone nanofibers, resulting in antimicrobial activity and eradication of *P. aeruginosa*. These studies highlighted how immobilization strategies can influence phage stability and antibacterial efficacy. Furthermore, Kotturi et al. [25] used coaxial electrospinning to incorporate Mycobacteriophage Fulbright into PCL nanofibers, with PCL forming the shell and phage in the core, enhancing antimicrobial activity against *M. smegmatis* and showing potential for phage-based treatment of various infections. Similarly, Kielholz et al. [16] employed a coaxial electrospinning approach to create fibers with a polyvinylpyrrolidone (PVP) polymer shell, encapsulating bacteriophages in the core while maintaining their antimicrobial activity against *S. aureus* and *P. aeruginosa*. Ju et al. [9] recently investigated phage-loaded electrospun fibers for treating wounds infected with *P. aeruginosa*. Anti–*P. aeruginosa* Neko phages were incorporated into both monoaxial and coaxial fibers. Phages were blended with polyvinyl alcohol (PVA) and processed either directly into fibers or as the core in coaxial electrospinning with PVP or PVP/ethyl cellulose (EC) shells. The addition of sucrose further improved phage viability, and coaxial PVA/Su–PVP fibers showed the highest retention of active phages. Furthermore, Suchithra et al. [26] made a dual-phage-integrated electrospun PVA-eudragit bilayer nanofiber matrix by incorporating PseuPha1 and RuSa1, targeting multidrug-resistant *P. aeruginosa* and *S. aureus*, via electrospinning, and evaluated its stability, antibacterial activity, and wound-healing potential.

Although electrospun nanofibers have previously been explored for phage delivery, several challenges still require further investigation, including low post-electrospinning phage viability and rapid, uncontrolled burst release. For example, monoaxial electrospinning studies have reported post-processing viabilities as low as 1–6% for bacteriophages such as T4, T7, and λ encapsulated in PVA fibers [27]. Similarly, burst release behavior has been observed in electrospun systems, with near-complete phage release occurring within less than one hour in certain coaxial fiber designs [28].

The present study introduces a novel PVA/PCL-gelatin nanofibrous dressing designed and developed for the delivery of *S. lugdunensis* phages. This pathogen has received limited attention despite its growing clinical relevance in skin and wound infections. To the best of our knowledge, no studies have systematically formulated and designed fiber-based systems optimized for phage stability and therapeutic efficacy, particularly against clinically relevant wound pathogens. The proposed dressing exhibits multifunctionality derived from both its material composition and hierarchical structure. The hydrophilic PVA layer, a water-soluble polymer used as a carrier for phages, is designed to dissolve immediately upon interaction with wound exudate, enabling immediate phage release and maintaining a moist wound environment. Meanwhile, the mechanically resilient PCL-gelatin support layer provides structural integrity, sustains phage release, and promotes cell adhesion through the presence of gelatin. Furthermore, this study uniquely compares long-tailed *E. faecalis* phage and novel short-tailed *S. lugdunensis* phages to assess how phage morphology affects encapsulation stability and antibacterial performance. Collectively, these advances establish a new functional and biological framework for phage-based wound dressings targeting *S. lugdunensis* infections, supported by comprehensive physicochemical and biological characterizations.

## 2. Materials and Methods

### 2.1. Chemicals

Poly (ε-caprolactone) (PCL, Mw = 80,000), Gelatin (from bovine skin, Type (B)), Polyvinyl alcohol (PVA, Mw = 85,000–124,000, 87–89% hydrolysed), 2,2,2-Trifluoroethanol (TFE, ≥99%), Glacial acetic acid (≥99%), and Glycerol (for molecular biology, ≥99%) were all purchased from Sigma-Aldrich (Sigma-Aldrich Co., St. Louis, MO, USA). Tween^®^ 80 (polysorbate 80; BDH Prolabo, VWR International S.A.S., Fontenay-sous-Bois, France) was also used in this study. Phosphate-buffered saline (PBS, pH 7.4) was prepared by dissolving sodium chloride (NaCl; Chem Supply Pty Ltd., Gillman, SA, Australia; 8.0 g/L), potassium chloride (KCl; Chem Supply Pty Ltd., Gillman, SA, Australia; 0.2 g/L), disodium phosphate dihydrate (Na_2_HPO_4_·2H_2_O; Sigma-Aldrich Co., St. Louis, MO, USA; 1.78 g/L), and monopotassium phosphate (KH_2_PO_4_; Sigma-Aldrich Co., St. Louis, MO, USA; 0.24 g/L) in distilled water. The pH was adjusted to 7.4, and the final volume was brought to 1 L.

### 2.2. Bacterial Culture and Phage Propagation

#### 2.2.1. Bacterial Strains and Culture Media

*Enterococcus faecalis ATCC* 700802 (American Type Culture Collection; ATCC, Manassas, VA, USA) was used for the isolation and propagation of the APTC-Efa.20 bacteriophage as previously described [29]. *S. lugdunensis* clinical isolates were obtained from chronic rhinosinusitis (CRS) patients’ nasal swabs. Bacterial swabs were collected after obtaining informed consent with protocols approved by the Central Adelaide Local Health Network Human Research Ethics Committee in Adelaide, South Australia, reference number (HREC/15/TQEH/132). Swabs were placed in a sterile Amies transport medium (Sigma Transwab, MWE Medical Wire, Corsham, UK) and transported to the laboratory for processing and identified using matrix-assisted laser desorption/ionization time-of-flight mass spectrometry (MALDI-TOF MS) at SA Pathology, employing a Bruker MALDI Biotyper system (Bruker Daltonics GmbH, Billerica, MA, USA).

Tryptic Soy Broth (TSB) and Bacteria Agar were purchased from Oxoid, Thebarton, SA, Australia. Magnesium Sulphate, Sodium Chloride, and Tris-HCL and polyethylene glycol (PEG 8000) were obtained from Sigma-Aldrich Co., St. Louis, MO, USA.

#### 2.2.2. Phage Isolation and Purification

Wastewater samples, obtained from the wastewater treatment plant at Christies Beach, Adelaide, SA, Australia, were centrifuged at 4000× *g* at 4 °C followed by filter sterilization using 0.22 µm filter (Acrodisc^®^, Pall Corporation, New York, NY, USA). Bacteriophage isolation and purification were performed as previously described [29]. Initially, phage was isolated using double-layer agar (DLA) where 500 µL of purified wastewater samples were mixed with a 100 µL of overnight culture of *S. lugdunensis* for 15 min in a glass tube. Four millilitres of 0.4% TSB molten agar were added to the mixture then poured into preprepared TSA plates and incubated overnight at 37 °C. Following incubation, clear plaques were picked using sterile pipette tips and stored aseptically in 1 mL SM buffer (100 mM NaCl, 8 mM MgSO_4_, and 50 mM Tris-HCl, pH 7.5). Following phage amplification [29], phages were extracted by gently mixing crude lysate with a precipitating solution (10% PEG-8000, 1 M NaCl) in 1:2 ratio. The mixture was kept at 4 °C to allow phage extraction. Following incubation, the mixture was centrifuged at 4000× *g* to pellet the phages and the supernatant was discarded. The phage pellets were suspended in 5 mL SM buffer and gently mixed with equal volume of chloroform to remove PEG from solution. The mixture was centrifuged at 4000× *g* for 10 min and the aqueous layer containing pure bacteriophages was collected and phage titer was calculated using DLA method as described above. The phage then stored at 4 °C for further use.

#### 2.2.3. Phage DNA Extraction and Sequencing

Phage DNA was extracted following the manufacturer’s guidelines using the Phage DNA Isolation Kit (Norgen Biotek Corp., Thorold, ON, Canada). To further purify the DNA, AMPure XP beads (Beckman Coulter Life Sciences, Pasadena, CA, USA) were employed, and DNA was quantified with a Qubit 3.0 fluorometer (Invitrogen, Life Technologies, Löhne, Germany), using the AccuGreen™ dsDNA Quantification Kit (Biotium Inc., Fremont, CA, USA).

Sequencing was done using an Oxford Nanopore Technologies MinION Mk1B, employing the RBK114.96 kit and an R10.4.1 chemistry flowcell (Oxford Nanopore Technologies, Oxford, UK). Dorado v0.8.0 with the dna_r10.4.1_e8.2_400bps_sup@v4.2.0 basecaller model was used to perform base calling. For sequence assembly and annotation, Sphae v1.3.2 [30] was utilized. Genome was assembled with Flye v2.9.3 [31] and subsequently reoriented to start with the predicted large terminase subunit using Dnaapler v1.2.0 [32]. Annotation was carried out with Pharokka v1.6.1 [33] and Phold v0.1.4 [34]. Phold leverages the ProstT5 protein language model [35] to align protein structural 3Di tokens with Foldseek v9-427df8a [36], searching against a phage protein structure database with over 1 million entries, generated using Colabfold [37] from the PHROGs [38] database.

#### 2.2.4. Host Range Analysis

The infectivity of the phage against *S. lugdunensis* clinical isolates was assessed using the double-layer spot assay (DLSA) as previously described [39]. In brief, 100 μL of *S. lugdunensis* culture (1.5 × 10^8^ CFU/mL) was combined with 4 mL of molten 0.4% tryptic soy broth (TSB) agar and poured onto solidified 1.5% TSB agar plates. Phage lysate (5 μL, at 1 × 10^6^ PFU/mL) was spotted onto the surface, with SM buffer used as a negative control. Plates were incubated at 37 °C overnight, and phage infectivity was evaluated based on the formation of lysis zones. Isolates were classified as sensitive (clear spot), semi-sensitive (turbid spot), or resistant (no lysis) [40].

#### 2.2.5. Temperature and pH Stability Assessment

Phage stability was evaluated across a range of temperatures (4 °C, 25 °C, 40 °C, 60 °C, 80 °C, and 100 °C) and pH values (2, 3, 5, 6, 7, 8, 9 and 11). Phage lysates (10^9^ PFU/mL) suspended in SM buffer were incubated under each condition for 1 h. Following treatment, samples were subjected to serial dilutions and phage titer was calculated using DLSA method as described above. After overnight incubation at 37 °C, phage viability was determined by counting the resulting plaques.

### 2.3. Polymer Solution and Electrospinning Process

#### 2.3.1. First Layer

PCL (≈12% *w*/*v*) and gelatin (≈12% *w*/*v*) solutions were separately prepared by dissolving the polymers in TFE at ambient temperature for approximately 3 h. Subsequently, the PCL and gelatin solutions were mixed at a weight ratio of 7:3. Finally, acetic acid (0.2% *v*/*v*) was added, and the mixture was stirred until a transparent and homogeneous solution was obtained. The solution was loaded into a 10 mL syringe with the required volume. Using silicone tubes, the syringe is connected to a 23-gauge needle, made of stainless steel. The solution was fed at a flow rate of 0.35 mL/h, using a syringe pump. The distance between the spinneret tip and the aluminium plate collector was maintained at 19 cm, and a voltage of 12 kV was applied. For comparison with PCL-Gel electrospun fibers, PCL solution was also electrospun under controlled parameters (flow rate: 0.8 mL/h, distance: 19 cm, voltage: 14 kV) for the same duration. To remove any residual solvents, the fibrous membranes were dried in a vacuum oven at 35 °C for 24 h.

#### 2.3.2. Second Layer

An aqueous PVA solution (≈8% *w*/*v*) was prepared by dissolving the polymer in distilled water under continuous stirring at 80 °C for 4 h. After cooling to ambient temperature, Tween 80 (1% *v*/*v*) and glycerol (4% *v*/*v*) were added to the PVA solution and the mixture was stirred for 1 h. Subsequently, just before the electrospinning process, bacteriophages in SM buffer were added to the solution at a weight ratio of 5:1. The solution was then delivered at a flow rate of 0.4 mL/h using the same needle as for the first layer, with a tip-to-collector distance of 9 cm and an applied voltage of 11–13 kV. The formulations and preparation procedures for electrospinning solutions, along with the corresponding electrospinning parameters, are summarized in Table 1.

**Figure 1:**
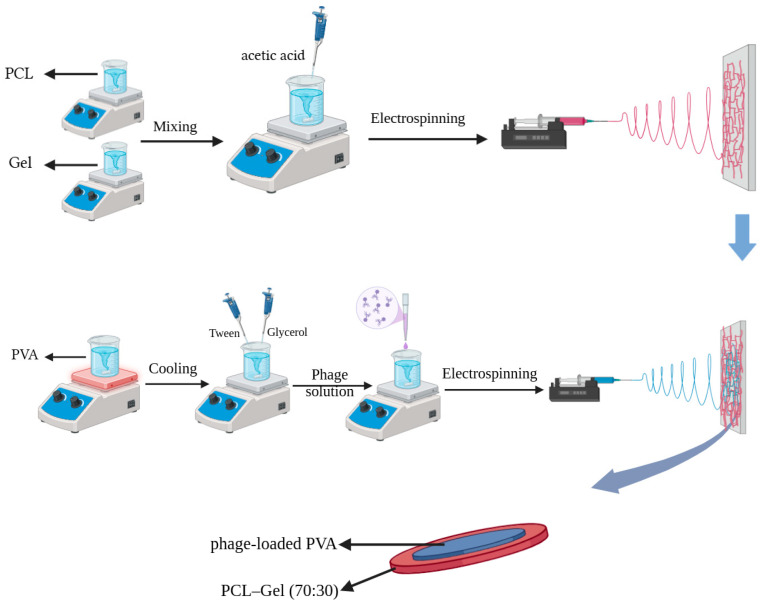
Schematic representation of the preparation of electrospinning solutions and the electrospinning process. The scheme was generated through BioRender.com (accessed on 30 September 2025).

Additionally, a schematic representation of the solution preparation and electrospinning process is presented in Figure 1.

### 2.4. Characterizations of Electrospun Fibers

#### 2.4.1. X-Ray Diffraction (XRD)

The phase identification of fibers was analyzed by X-ray diffraction (XRD) using a Rigaku MiniFlex 600 diffractometer (Rigaku Corporation, Tokyo, Japan) equipped with Cu Kα radiation (λ = 1.54178 Å), operated at 40 kV and 15 mA. The diffraction patterns were collected at a scanning rate of 10° min^−1^ with the step sizes of 0.02° in the range 5° < 2θ < 45°.

#### 2.4.2. Fourier Transform Infrared (FTIR) Analysis

To characterize the composition of the fibers, the uniaxial fibers were directly placed on the ATR (Attenuated Total Reflection) module of a Nicolet iS50 ATR-FTIR spectrometer (Thermo Fisher Scientific Inc., Madison, WI, USA) with a scan range of 500 to 4000 cm^−1^ and recorded with a resolution of 4 cm^−1^ by 32 scans.

#### 2.4.3. Thermal Stability

To investigate the thermal decomposition of the nanofibers, thermogravimetric analysis (TGA) performed on a Netzsch STA 449 F5 Jupiter thermal analyzer (NETZSCH-Gerätebau GmbH, Selb, Germany). Approximately 7.5 mg of fibers were cut and placed in an Al_2_O_3_ crucible, and the measurements were carried out from 25 °C to 700 °C at a heating rate of 5 °C min^−1^ under a nitrogen flow of 50 mL min^−1^.

#### 2.4.4. Morphology and Surface Characterizations

The morphological characteristics of the fibrous membranes were examined using both optical and scanning electron microscopy (SEM). A Nikon Eclipse Ti inverted optical microscope (Nikon Corporation, Tokyo, Japan) provided a general view of the fibers, while a Hitachi SU7000 SEM (Hitachi High-Tech Corporation, Tokyo, Japan) was used for detailed surface analysis at an accelerating voltage of 5 kV. Nanofiber specimens were mounted on metallic stubs using double-sided adhesive tape and sputter-coated with platinum in an argon atmosphere for approximately 40 s, achieving a coating thickness of ~5 nm. The Image J software (Fiji, version 1.54p) was employed to assess the mean diameter and frequency distribution of each fiber specimen. Approximately 60 fibers from the SEM image were randomly selected for this analytical purpose.

Transmission electron microscopy (TEM) was conducted using a FEI Tecnai G2 Spirit microscope (FEI Company, Hillsboro, OR, USA) operated at an accelerating voltage of 120 kV to investigate both the bacteriophages and the second fiber layer incorporating them. Samples for the second layer were prepared by directly spinning onto carbon/formvar-coated 300-mesh copper grids (ProSciTech Pty Ltd., Kirwan, QLD, Australia) for a few seconds. For phage imaging, 1 mL aliquots of high-titre lysate (10^9^ PFU/mL) were used as described previously [41]. Briefly, 5 μL of the phage sample was applied to the grids and allowed to incubate for 3 min before being blotted dry. The sample was then sequentially treated with 5 μL of TEM fixative (1.25% glutaraldehyde, 4% paraformaldehyde in PBS with 4% sucrose) for 2 min, followed by 5 μL of 2% uranyl acetate for 2 min.

Wettability (Water contact angle) was determined by measuring the contact angle of the fiber films using an optical tensiometer (Attension T200, Biolin Scientific AB, Gothenburg, Sweden) with a droplet volume of 5 microliters. Each measured sample underwent testing three times, and the resulting average value was computed.

#### 2.4.5. In Vitro Degradation Test

For the in vitro degradation study, nanofiber mats were cut into square-shaped specimens and incubated in phosphate-buffered saline (PBS, pH 7.4) at 37 °C for up to seven days (sampling times: 3, 6, 12, 18, 24, 48, 72, and 168 h). At each predetermined time point, the samples were removed, rinsed with distilled water, and dried in a vacuum oven at 37 °C for 24 h. The dried specimens were then weighed, and the percentage of weight loss was calculated using Equation (1), where *W_i_* and *W_t_* represent the initial and remaining weights, respectively.

(1) $$Degree\;of\;degradation\;\left(\%\right)=\frac{W_{i}-W_{t}}{W_{i}}\times 100\%$$

### 2.5. Biological and Functional Assessments

#### 2.5.1. Antimicrobial Performance Assessment

The antibacterial activity of the loaded nanofibers was tested using DLA method. Briefly, 100 µL of either *S. lugdunensis* or *E. faecalis* bacterial suspensions (1.5 × 10^8^ CFU/mL) were mixed with 4 mL of 0.4% *w*/*v* molten agar and poured into 1.5% *w*/*v* TSA agar plates and allowed to solidify. Nanofibers were cut into different shapes with and without aluminium foil and applied to the top agar layer containing bacteria and incubated at 37 °C for 24 h. Following incubation, plates were photographed to demonstrate antibacterial activity of the released phages from the nanofibers compared to blank nanofibers without phages.

#### 2.5.2. Viability of Phages in Electrospun Fibers

The viability of bacteriophages in nanofibers was determined using the published protocol [9]. In a 50 mL Falcon tube, nanofiber sheet (approximately 10 mg of monoaxial fibers) loaded with phages was immersed in 5 mL SM buffer (pH 7.5) and incubated at 37 °C under shaking at 120 RPM. 1 mL aliquots of the buffer solution were subjected to serial dilution in SM buffer and phage titre was determined using DLSA method and the total amount of released phages in 5 mL was quantified. The phage titre in the polymer mixture was determined, and the amount of the loaded phage was quantified per mg of fibers. The viability of phages in the fibers was determined according to the equations:

Viability % = Viable Phage released from fibres/Total phage loaded in fibres × 100(2)

This measurement reflects the fraction of phages retaining infectivity after electrospinning.

#### 2.5.3. In Vitro Phage Release Profile

The release kinetics from the nanofibers was determined as described with modifications [28]. Nanofiber sheet containing bacteriophage was incubated in 5 mL SM buffer at 37 °C under shaking at 120 RPM; aliquots of 100 µL were drawn at different time intervals and replaced with fresh 100 µL SM buffer. The withdrawn samples were serially diluted, and titres were determined using DLSA. Phage release profiles were obtained by plotting log_10_ (cumulative PFU) against time.

### 2.6. Statistical Analysis

The data are presented as the mean ± SD. GraphPad Prism (version 10; GraphPad Software, La Jolla, CA, USA) was used for graphing and statistical analysis. Differences between groups were assessed using One-way Analysis of Variance (ANOVA) followed by multiple comparisons Tukey’s test, where *p*-value < 0.05 was considered statistically significant; * *p* < 0.05; ** *p* < 0.01; *** *p* < 0.001 and **** *p* < 0.0001.

## 3. Results

### 3.1. Structural and Composition Characterizations of Prepared Electrospun Fibers

**Figure 2:**
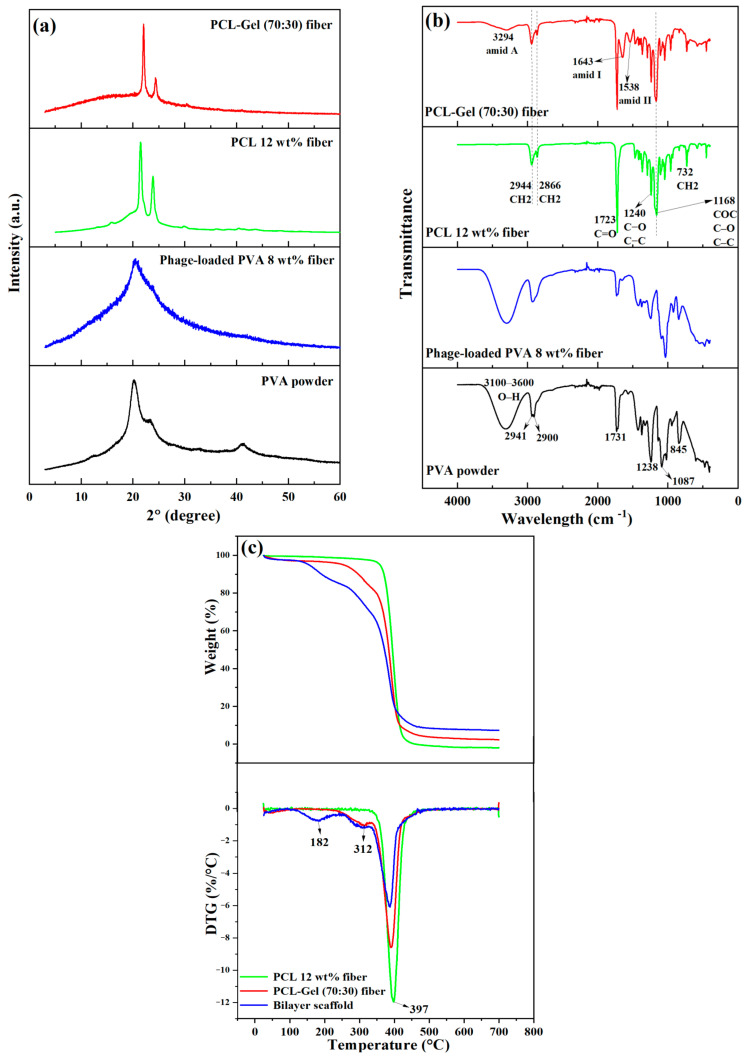
(**a**) XRD patterns; (**b**) FTIR spectra of PVA powder, phage-loaded PVA, PCL, and PCL-Gel (70:30) nanofibers, and (**c**) TG and DTG curves of PCL, PCL-Gel (70:30) nanofibers, and the bilayer scaffold.

The XRD patterns of PVA powder, phage-loaded PVA, PCL, and PCL-Gel (70:30) nanofibers are presented in Figure 2a. For comparison, the XRD patterns of PVA powder and PCL fibers are included to better identify the characteristic diffraction peaks. Both pristine samples exhibit the typical semi-crystalline nature of polymers, with amorphous regions observed at 2θ ≈ 10–30° for PVA powder and 2θ ≈ 15–20° for PCL fibers, along with distinct crystalline features. PVA powder shows characteristic diffraction peaks at 2θ ≈ 20.18° and at 2θ ≈ 41.26°. After electrospinning, however, the XRD pattern of PVA fibers displays a broad peak centered at 2θ ≈ 20.5°, corresponding to the (101) crystal reflection plane, which suggests a change in crystallinity. In the case of PCL, the two most intense peaks appear at 2θ ≈ 22° and 2θ ≈ 24°, corresponding to the (110) and (200) crystal planes, respectively. The diffraction positions mentioned for the samples are in good agreement with previously reported studies [42,43,44,45,46,47,48,49]. Gelatin, as a protein-based material, exhibits a broad diffraction halo at 2θ ≈ 10–20°, indicative of its amorphous nature [50,51]. This feature is also evident in the XRD pattern of PCL-Gel (70:30) fibers in Figure 2a.

The FTIR spectra pertaining to the powder and nanofibers are illustrated in Figure 2b. The FTIR spectrum of pure PVA powder exhibited a broad absorption band between 3600 and 3100 cm^−1^, attributed to hydroxyl (O–H) stretching vibrations from intermolecular and intramolecular hydrogen bonds. In addition, distinct peaks were observed at 2941 and 2900 cm^−1^ (–CH_2_ asymmetric and symmetric stretching), 1731 cm^−1^ (C=O stretching of residual acetate groups), 1238 cm^−1^ (C–H and –CH_2_ stretching), 1087 cm^−1^ (C–O stretching of acetyl groups), and 845 cm^−1^ (C–C vibrations). These assignments are consistent with previous reports [52,53]. No distinct differences were observed between the FTIR spectra of PVA powder and phage-loaded PVA fibers. That is, after the addition of glycerol and electrospinning, the molecular composition and functional groups remained nearly unchanged, which is consistent with a previous report [54]. Moreover, the stretching band corresponding to C=O of carbonyl esters was identified at 1723 cm^−1^ within the pure PCL nanofibers. The band associated with the crystalline phase of PCL at 1240 cm^−1^ was designated to C–O and C–C stretching, while the peak at 1168 cm^−1^ was ascribed to the vibrations of COC, C–O, and C–C. Furthermore, the peak at 732 cm^−1^ denoted the CH_2_ bending of the caprolactone chain, and the peaks at 2866 cm^−1^ and 2944 cm^−1^ exhibited symmetric and asymmetric CH_2_ stretching, respectively. All the peaks of PCL mentioned above are well aligned with the literature [55,56]. In the FTIR spectra of the PCL-Gel (70:30) nanofibers, the characteristic bands associated with gelatin appeared at 1538 cm^−1^ (coupling of N-H bending vibration and C–N stretching vibration—amide II), 1643 cm^−1^ (C=O stretching vibration in peptide bonds—amide I), and 3294 cm^−1^ (N–H and O–H vibrations—amide A) [57,58,59].

As shown in Figure 2c, the thermal decomposition behaviour of the fibers was evaluated using thermogravimetry (TG) and derivative thermogravimetry (DTG). Pure PCL (12 wt%) fibers exhibited a single-step degradation profile, whereas the introduction of additional phases increased the number of decomposition steps, corresponding to the degradation of each component. Specifically, PCL-Gel (70:30) fibers displayed two distinct steps, while the bilayer scaffold containing phage-loaded PVA (8 wt%) exhibited three steps. Although the ending temperatures (T_e_) of all samples were nearly identical, the initial decomposition temperature (T_i_) varied. PCL (12 wt%) showed the highest T_i_ (~335 °C), whereas the bilayer scaffold exhibited the lowest T_i_ (~114 °C), followed by gelatin degradation at ~220 °C. The temperature at the maximum weight loss rate (T_m_) was almost similar for all samples, ranging from 387 to 397 °C, and is associated with PCL degradation, with the highest T_m_ observed in pure PCL. In PCL-Gel (70:30), the first peak appeared at ~312 °C, which closely corresponds to the second peak of the bilayer scaffold, both attributed to gelatin degradation. Additionally, the first DTG peak of the bilayer scaffold occurred at ~182 °C, corresponding to PVA degradation. The results are comparable with those of previous studies [50,60]. Moreover, the onset temperature of PCL degradation in the PCL-Gel (70:30) and bilayer scaffolds was comparable to that of pure PCL (12 wt%), occurring after approximately 20% and 30% weight loss, respectively. The residual ash content was estimated at 2.4% for PCL-Gel (70:30) and 7.4% for the bilayer scaffold.

**Figure 3:**
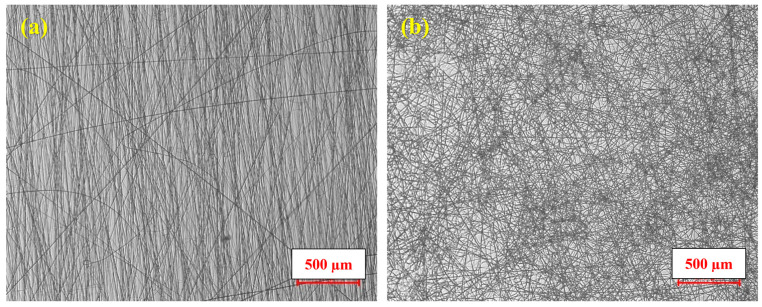
Optical microscope images of (**a**) PCL-Gel nanofibers, and (**b**) phage-loaded PVA nanofibers at 20× magnification.

For the morphological and surface characterizations of the fibers, optical microscopy, SEM, WCA measurement, and TEM were employed. In addition, degradation behavior was evaluated through degradation testing and determination of the degree of degradation. Figure 3 shows optical microscope images of PCL-Gel and phage-loaded PVA nanofibers, which were captured to give a general view of the fibers. Figure 3a shows fibers with a tendency to align along straight directions, whereas Figure 3b shows a randomly oriented fibrous structure with an entangled network.

**Figure 4:**
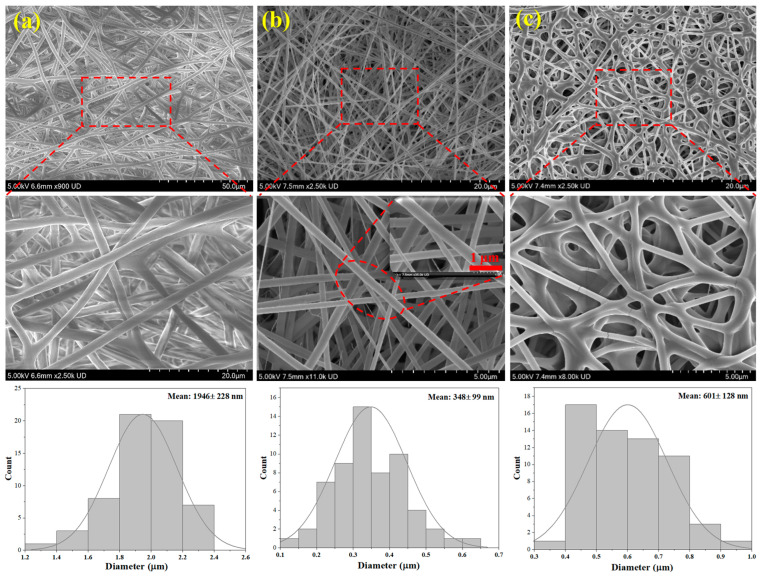
SEM micrographs of fibers, along with their diameter distribution histograms of (**a**) pure PCL fibers; (**b**) PCL-Gel nanofibers, and (**c**) phage-loaded PVA nanofibers.

According to Figure 4, SEM images of the electrospun fibers at different magnifications were analyzed to examine the morphologies in greater detail. Both pure PCL and PCL/Gel electrospun fibers were predominantly straight, whereas the phage-loaded PVA nanofibers formed a network of entangled fibers with multiple branches, consistent with the observations from optical microscopy. As illustrated, the surface morphology of the fiber mats predominantly exhibited non-aligned, smooth, and uniform arrangements, with flawless polymer fibers. The average fiber diameter of the PCL/Gel mats decreased to 348 ± 99 nm upon the incorporation of gelatin, compared to 1946 ± 228 nm for the pure PCL fibers. In addition, the phage-loaded PVA nanofibers, serving as the second layer, exhibited an average fiber diameter of 601 ± 128 nm as shown in the diameter distribution histograms. Ju et al. [9] fabricated monoaxial fibers using a PVA solution containing sucrose and phage suspension, achieving an average fiber diameter of approximately 742 nm. Similarly, Sarhan and Azzazy [22] produced monoaxial electrospun fibers composed of PVA, honey, chitosan, bee venom, and phages, with an average diameter of around 600 nm. The average diameter of the phage-loaded PVA nanofibers obtained in the present study is therefore consistent with these previously reported findings.

**Figure 5:**
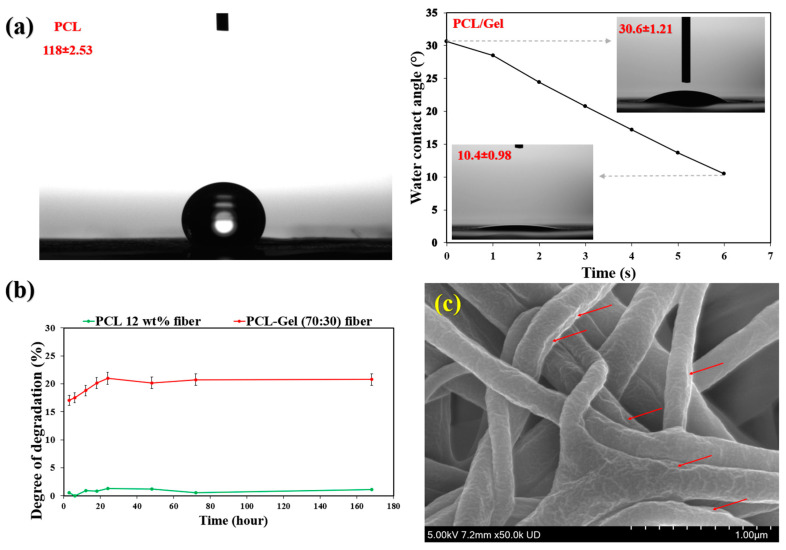
(**a**) The water contact angle; (**b**) degree of degradation of pure PCL and PCL/gelatin fibers, and (**c**) SEM micrograph of PCL/Gel after 24 h degradation.

PCL fibers exhibit hydrophobic characteristics due to the presence of methylene (–CH_2_–) groups in their main chain, with reported water contact angles ranging from approximately 114° to 130° [43,44,46,50,61]. As shown in Figure 5a, the pure PCL fibers demonstrated a contact angle of around 119°, confirming their hydrophobic nature. However, the incorporation of gelatin significantly enhanced the wettability of the PCL/Gel electrospun fibers, reducing the water contact angle to approximately 30.6°, which further decreased to about 10.49° after 6 s (Figure 5a). This remarkable increase in wettability can be attributed to the hydrophilic nature of gelatin, a biocompatible and biodegradable protein rich in polar functional groups that promote water absorption [1]. This high wettability suggests that the PCL/Gel electrospun mat is well suited as the first layer, as it can readily absorb wound exudates and provide a favourable substrate for the subsequent release of phages from the second layer. Furthermore, the phage-loaded PVA nanofibers exhibited a highly hydrophilic surface, indicating excellent wettability. This behaviour can be attributed to the presence of glycerol in the electrospinning solution and the shorter electrospinning duration (20 min), which resulted in a very thin second layer and, consequently, very fast dissolution and degradation upon contact with moisture.

The degradation behaviour of PCL (12 wt%) and PCL-Gel (70:30) nanofibers is presented in Figure 5b. Pure PCL exhibited negligible weight loss throughout the study. In contrast, PCL-Gel (70:30) nanofibers showed a degradation of approximately 17% after 3 h of incubation in PBS, which increased to about 20% at 24 h. Beyond this point, the degradation remained nearly constant. According to the SEM micrograph of PCL/Gel fibers after 24 h of degradation (Figure 5c), the fibers appeared less smooth and uniform compared to those in Figure 4b. Surface erosion and degradation features are clearly visible along the fiber surfaces, as indicated by the arrows. It should be noted that degradation was evaluated in PBS, which does not fully replicate the enzymatically active wound environment. PBS was selected to provide a controlled baseline and to maintain consistency with the biological assays performed at physiological pH and temperature, enabling clearer interpretation of the influence of phage morphology on scaffold performance.

**Figure 6:**
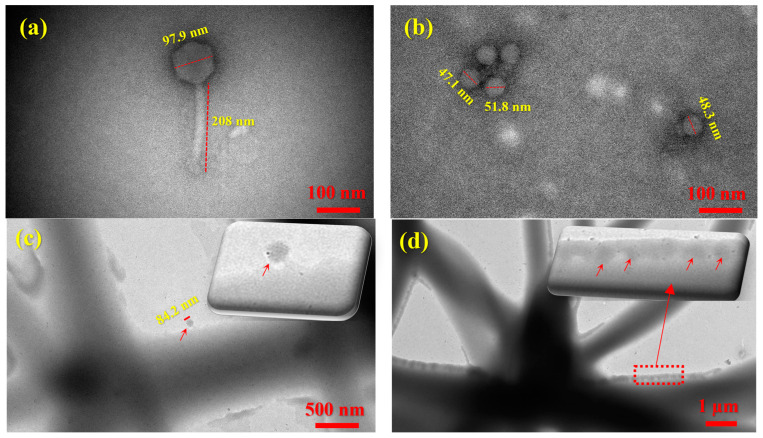
TEM images of (**a**) a long-tailed phage; (**b**) short-tailed phages; (**c**) PVA fibers loaded with long-tailed phages, with small red arrows indicating a protruding long-tailed phage outside the fiber matrix; and (**d**) PVA fibers loaded with short-tailed phages, with small red arrows highlighting phages encapsulated within the fibers.

Figure 6 presents TEM images of the bacteriophages used in this study, along with carrier fibers encapsulating both long- and short-tailed phages. The TEM image of the long-tailed phage APTC-Ef.20 (Figure 6a) shows its characteristic icosahedral head and contractile tail morphology, typical of the myovirus morphology, with an average capsid diameter of 97.9 ± 2.5 nm and a tail length of 208 ± 5.0 nm. In contrast, the TEM image of the short-tailed phage (APTC-SL.1) displays a podovirus morphology, characterized by a short, non-contractile tail and an icosahedral head (Figure 6b), with an average capsid diameter of 48.8 ± 2.2 nm and a tail length of 32.6 ± 2.8 nm. Due to the relatively large diameters of the carrier fibers and the presence of excipients, the contrast between the polymer matrix and the embedded phages was not sufficient for clear visualization, a limitation also reported in previous studies [27]. As shown in Figure 6c, a long-tailed phage can be observed outside a fiber, likely expelled during processing. In contrast, Figure 6d reveals short-tailed phages clearly visible within the carrier fiber matrix due to the relatively good contrast at the fiber edges. Multiple virions appear anchored along the fiber boundary, confirming the successful incorporation of Podoviridae-like particles into the delivery scaffold.

### 3.2. Biological Characterizations of the S. lugdunensis Phage

#### 3.2.1. Genome Sequencing

The phage APTC-SL.1 belongs to the *Rountreeviridae* family of the Andhravirus genus and possesses a double-stranded DNA genome measuring 18,292 base pairs, with a GC content of approximately 30%. Bioinformatic analysis revealed 20 predicted coding sequences (CDS), of which 13 could be assigned putative functions. APTC-SL.1 showed no evidence of lysogeny-related genes, indicating a strictly lytic lifecycle. Additionally, genomic screening using Pharokka against the CARD antimicrobial resistance database [62] and the VFDB virulence factor database [63] confirmed the absence of known toxin genes, antibiotic resistance genes, and virulence factors.

#### 3.2.2. Host Range

A collection of 10 *S. lugdunensis* clinical strains isolated from CRS patients were tested for sensitivity against APTC-SL.1. The phage was able to infect 50% of the strains. The details of host range analysis are shown in Table 2. 

#### 3.2.3. Stability of APTC-SL.1 at Various Temperatures and PH Levels

**Figure 7:**
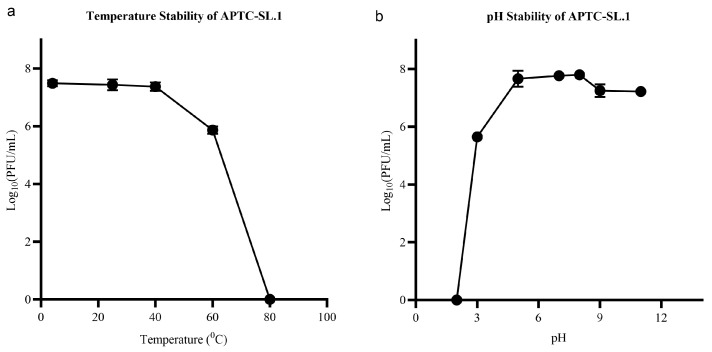
Stability studies of APTC-SL.1. (**a**) represents phage titres following 1 h incubation at different temperatures. (**b**) represents phage titres following 1 h incubation in SM buffer at different pH values.

The phage viability remained stable after 1 h of incubation at temperatures (4–40 °C) with no significant change in titre compared to the control incubated at 37 °C (*p* = 0.8092). A significant (~2 log_10_) reduction in phage titre was observed following incubation at 60 °C (*p* < 0.0001), and no viable phages were detected at 80 °C or higher (Figure 7). In terms of pH stability, the phage titre did not significantly change between pH 5 and 11 (*p* < 0.05), while (~2 log_10_) reduction in viability was recorded at acidic pH 3 (Figure 7). These data indicate the stability of the phage at a wide range of temperatures and pH values used in the propagation process. A similar stability behaviour of the myovirus phage APTC-EfA.20 was previously reported. APTC-Efa.20 is resilient to changes in pH and temperature, with no reduction in phage titre between pH 4–10 and high temperatures up to 60 °C [29].

### 3.3. Antimicrobial Performance of S. lugdunensis Phage-Loaded Electrospun Fibers

#### 3.3.1. Antibacterial Activity Assessment

**Figure 8:**
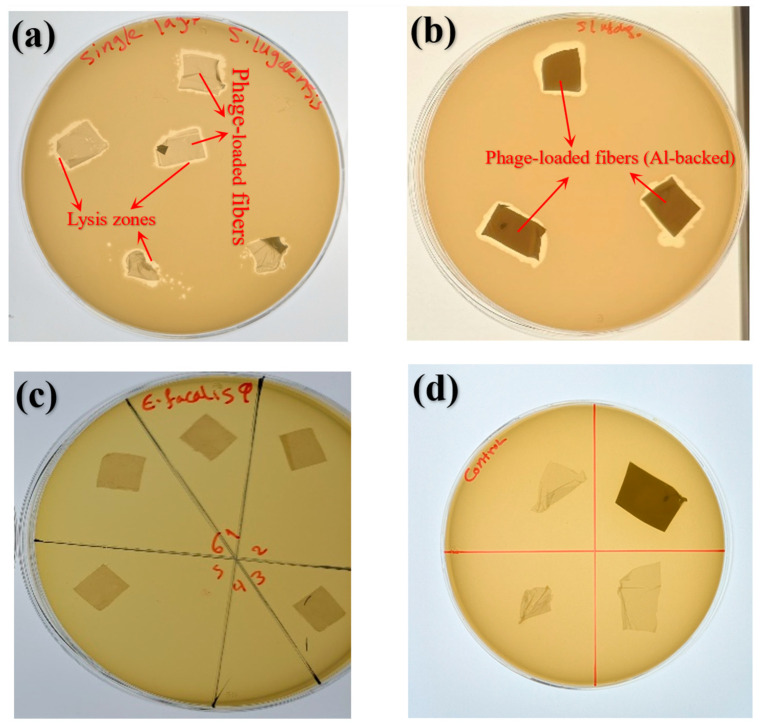
Antibacterial activity of bilayer nanofiber scaffolds. (**a**,**b**) Nanofibers loaded with APTC-SL.1 showing clear lysis zones around the fibers, indicating successful phage release and antibacterial activity; (**c**) nanofibers loaded with APTC-Efa.20 showing no lysis zones around the fibers, and (**d**) control nanofibers without phage loading.

The bilayer scaffold wound dressing, composed of a supportive PCL/gelatin base layer and a PVA top layer loaded with bacteriophages, was evaluated against lawns of *S. lugdunensis* CI 1126 using an agar diffusion assay. After 24 h of incubation, clear lysis zones were observed, confirming the successful release and antimicrobial activity of short-tailed phages from the phage-loaded bilayer dressings of various shapes, both with and without aluminium foil (Figure 8a,b). In contrast, the control scaffolds exhibited no antimicrobial activity (Figure 8d). Moreover, the carrier fibers loaded with long-tailed phages APTC-Efa.20 did not exhibit antibacterial activity or lysis zones against the host bacterium ATTC 700802, as shown in Figure 8c. This lack of activity may be influenced by the structure and morphology of these phages, which could make them more vulnerable to the forces encountered during electrospinning. According to SEM images, the carrier fibers had an average diameter of about 601 nm (Figure 4c), whereas the long-tailed phages measured approximately 300 nm in length (Figure 6a). In contrast, the short-tailed phages, with an average size of about 81 nm (Figure 6b), were less exposed to the electrospinning forces, including the applied voltage, which likely contributed to their preserved activity. As shown in the TEM images (Figure 6c), a long-tailed phage was observed outside a fiber and exhibited morphological alterations, whereas no short-tailed phages were detected outside the fibers (Figure 6d), suggesting that short-tailed phages may be more effectively encapsulated and protected under harsh processing conditions, potentially due to their morphology and structure. Although direct evidence of tail breakage was not observed, the long-tailed phage imaged after electrospinning displayed notable deviations from its typical morphology, including apparent capsid deformation and a reduction in head size compared to its expected icosahedral structure, which may indicate structural stress induced during processing. Bacteriophages possess a complex architecture that can make them susceptible to damage under extreme fabrication conditions. Although capsids are generally considered more resistant to physical stress, tail structures, particularly tail fibers, have been reported to be more sensitive to mechanical forces [27]. Taken together, these observations support the hypothesis that long-tailed phages may be more vulnerable to the combined effects of electric field exposure, shear stress, and rapid dehydration during electrospinning, which could contribute to their reduced biological activity relative to short-tailed phages.

#### 3.3.2. Phage Viability After Electrospinning

Phage viability is a critical parameter that reflects the proportion of phages retaining infectivity after the electrospinning process. Since phage viability directly impacts therapeutic efficacy, minimizing titer loss during electrospinning is essential. In the present study, the viability of the bilayer scaffold loaded with short-tailed phages was determined to be 8.15 ± 0.7% according to Equation (2) (phage titer in the polymer stock prior to electrospinning: 5.4 × 10^8^ PFU/mL; phage titer in the nanofibers: 2.2 × 10^5^ PFU/mL), which is comparable to previously reported findings. For example, Salalha et al. [27] encapsulated different phages (T4, T7, and λ) in PVA monaxial fibers and observed post-electrospinning viabilities of 1%, 2%, and 6%, respectively. More recently, Ju et al. [9] investigated the use of alginate and sucrose (Su) as excipients in PVA monaxial fibers. They found that fibers without excipients retained only 3.55% phage viability, while PVA/alginate fibers showed a further reduction to 0.34%. In contrast, the addition of sucrose significantly improved outcomes, with viabilities of 7.31% in PVA/0.1 M Su fibers and 7.25% in PVA/0.5 M Su fibers. The authors emphasized that sucrose stabilizes phages by maintaining intraprotein hydrogen bonding and substituting for water molecules during dehydration in electrospinning. Similarly, in our study, glycerol likely contributed to phage protection by forming hydrogen bonds with proteins and water molecules, stabilizing their structure during dehydration. It also reduced dehydration-induced osmotic stress, preserving phage integrity during drying. Previous studies have also shown that glycerol can substantially improve the viability of living cells, likely by stabilizing their structure and reducing dehydration-induced stress [27].

It is worth mentioning that although the phage viability achieved in this study (~8.15%) may appear modest from a translational perspective, there is currently no established minimum effective phage dose specifically defined for electrospun fiber-based wound dressings. While topical phage therapy has demonstrated efficacy in several in vivo studies, only a limited number of reports have evaluated electrospun phage-loaded scaffolds in wound models [21,22], and effective doses vary widely depending on wound conditions, bacterial load, and phage type. Notably, viabilities of phages after electrospinning in the range of approximately 0–14% are commonly reported for electrospun phage-loaded systems, reflecting the inherent challenges associated with high-voltage processing and rapid dehydration. The observed phage viability in this study therefore falls within the reported literature range. Furthermore, the self-replicating nature of bacteriophages at the infection site, combined with the relatively sustained release behavior of the bilayer scaffold, may partially compensate for reduced initial viability. Strategies such as the incorporation of protective excipients, coaxial electrospinning, or further optimization of electrospinning parameters, such as those reported by Ju et al. [9], who achieved phage viabilities of up to ~14.5% using coaxial electrospinning and a suitable excipient.

#### 3.3.3. In Vitro Phage Release Profiles

**Figure 9:**
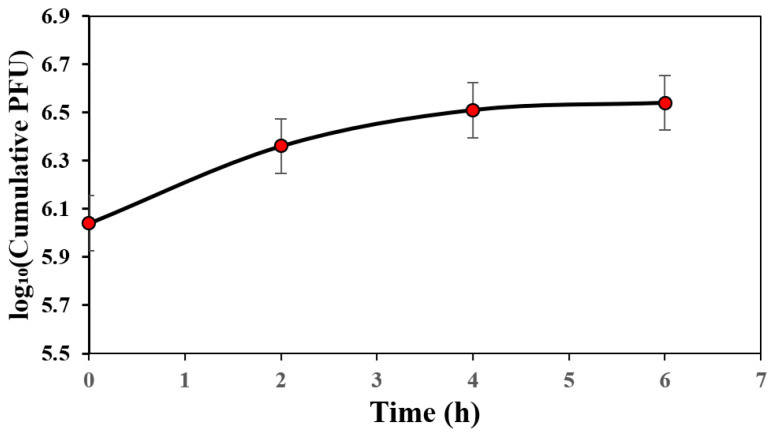
Phage release profiles from electrospun fibers loaded with the phage APTC-SL.1. Phage release is expressed as log_10_ PFU/mL over time (up to 6 h).

In Figure 9, the cumulative release profile of bacteriophages expressed as log_10_ (PFU) demonstrated an initial fast release within the first 2 h, where the concentration increased from 11 × 10^5^ PFU (log_10_ ≈ 6.04) at 0 h to 23 × 10^5^ PFU (log_10_ ≈ 6.36). Moreover, a slower but continuous release was observed between 2 and 4 h, reaching 32 × 10^5^ PFU (log_10_ ≈ 6.51). Beyond 4 h, the release approached a plateau, with only a slight increase to 35 × 10^5^ PFU (log_10_ ≈ 6.54) at 6 h, indicating that most of the loaded phages were released during the early stages, followed by a saturation phase.

Given the hydrophilic nature of the carrier polymer (PVA) and the characteristics of electrospun fiber mats (high porosity and large surface area), it is expected to have a burst release of phages as mentioned by other studies. Ju et al. [9] mentioned that both the PVA/Su + phage monoaxial fibers and the PVA/Su PVP + phage coaxial fibers containing a PVA/Su + phage core, with the addition of a PVP shell have similar burst phage release profile, given the hydrophilic nature of the polymers, suggesting that the PVP shell does not have any influence on the release. Similarly, in the study by Korehei and Kadla [64], the release rate was under 1 h as they encapsulated a T4 phage/buffer suspension within PEO and PEO/CDA shell fibers using coaxial electrospinning. However, as shown in Figure 9, the loaded phages were released within 6 h, showing much slower and relatively sustained release in cumulative PFU (5.9 to 6.5 log over 6 h) compared to literature. This phenomenon can be attributed to the structure and composition of the scaffold developed in this study. The bilayer design not only provides mechanical strength via the PCL layer but also contributes to a reduced and relatively sustained release of phages. This relatively sustained release likely arises from multiple mechanisms: upon exposure to moisture, gelatin in the underlying PCL/gelatin layer can degrade and dissolve (Figure 5c, red arrows), while the PCL/gelatin interfaces may adsorb phages through electrostatic interactions, hydrogen bonding, or nonspecific protein adsorption, collectively promoting relatively controlled release. Additionally, gelatin can stabilize the PVA outer layer by forming hydrogen bonds, enhancing its structural integrity, slowing dissolution, and further moderating phage release. Collectively, these findings, together with previous studies, emphasize that both the formulation and structural design of electrospun scaffolds are critical for enhancing and preserving phage viability, and achieving relatively controlled and continuous release, with phage viability of ~8.15%.

It should be noted that, as implied, the phage release profile observed in this study is dominated by an initial burst within the first few hours, which is consistent with the hydrophilic nature of PVA and the highly porous structure of electrospun fiber mats. A release plateau was reached within the studied timeframe, with no additional measurable phage release observed thereafter. While extended antimicrobial activity over days is often desirable for wound healing, rapid phage release may be beneficial during the early stages of treatment by reducing bacterial load and preventing infection establishment. Moreover, bacteriophage replication at the infection site may prolong antimicrobial activity beyond the initial release period. Nevertheless, the development of wound dressings capable of sustained multi-day phage release will require further optimization of scaffold design and is beyond the scope of the present study. From a design perspective, this release behavior could be modulated in future studies through strategies such as mild crosslinking of the PVA layer, blending PVA with less water-soluble polymers, or further optimization of multilayer scaffold architectures to slow polymer dissolution and enable more sustained phage release.

## 4. Conclusions

In this study, the biological activity of two bacteriophages with distinct morphologies, short-tailed podovirus and long-tailed myovirus, encapsulated within a bilayer electrospun scaffold was systematically investigated. The bilayer electrospun structure was fabricated by first electrospinning a PCL-Gel support layer, followed by deposition of a PVA top layer containing glycerol as an excipient and the encapsulated phages. Comprehensive physicochemical characterization using X-ray diffraction (XRD), Fourier transform infrared spectroscopy (FTIR), and thermogravimetric analysis (TGA) confirmed that the developed scaffolds possessed the appropriate structural integrity and chemical compatibility required for wound dressing applications. Morphological and surface analyses further demonstrated the formation of smooth, uniform fibers with excellent wettability and controlled degradation behavior attributed to the presence of gelatin. Biological assessments revealed that the short-tailed phage exhibited higher resistance to the electrospinning process, maintaining infectivity and producing clear zones of bacterial lysis against *S. lugdunensis*. Its approximately 8.15% viability may be associated with more effective encapsulation within the polymer carrier under the applied electrospinning conditions. The bilayer design also provided favorable in vitro release profiles, supporting relatively sustained phage delivery. While differences in host–phage interactions may also play a role, the observed trends underscore the importance of phage morphology and structure in the performance of electrospun scaffolds. Overall, these findings highlight the critical influence of phage morphology and structure, scaffold design, and material composition on the performance of phage-loaded electrospun dressings. The developed PVA/ PCL-Gel scaffold shows strong potential as a next-generation phage-based wound dressing. Future work should focus on optimizing fiber architecture and exploring strategies to enhance phage stability during electrospinning and long-term storage.

## Figures and Tables

**Table 1:** Formulations of electrospinning solutions and corresponding electrospinning parameters.

Formulations	Electrospinning Parameters				
	Voltage (kV)	Flow Rate (mL/h)	Distance (cm)	Time (mins)	Homogeneity (mm)
PCL 12%	14	0.8	19	105	30
PCL 12%/Gel 12%	12	0.35	19	105	30
PVA 8%/Tween 1%/Glycerol 4%/phage solution	11–13	0.4	9	20	45

**Table 2:** Host range analysis of APTC-SL.1 against *S. lugdunensis* clinical strains.

*S. lugdunensis* Clinical Strain	Sensitivity of APTC-SL.1
C1126	+ (Host)
C613	−
C631	+
C701	−
C734	+
C977	−
C987	−
C997	+/−
C1022	+
C1037	−
C1054	+
Host range	50%

+ = sensitive, +/− = semi-sensitive, − = non-sensitive.

## Data Availability

The data presented in this study is available on request from the corresponding author.

## Abbreviations

The following abbreviations are used in this manuscript:

Gel	Gelatin
PBS	phosphate-buffered saline
TSB	Tryptic Soy Broth
